# ‘But’ Implicatures: A Study of the Effect of Working Memory and Argument Characteristics

**DOI:** 10.3389/fpsyg.2016.01520

**Published:** 2016-11-08

**Authors:** Leen Janssens, Walter Schaeken

**Affiliations:** Laboratory for Experimental Psychology, Department of Brain and Cognition, KU LeuvenLeuven, Belgium

**Keywords:** conventional implicatures, but, working memory, automaticity, context

## Abstract

This study aimed to investigate the possible cognitive costs involved in processing the implicatures from *but* and the conclusion introducing words *so* and *nevertheless*. Adult participants were asked to indicate the conclusion that the person in the story would make, based on ‘p but q’ sentences constructed as indirect distancing contrasts. Additionally, while performing this task, participants’ working memory was burdened with a secondary dot recall task in four conditions ranging from no working memory load to high load. The results showed that working memory load did not influence participants’ performance on the implicature task. This finding might be interpreted to suggest that working memory is not involved in inferring the implicatures from *but*, *so*, and *nevertheless*. We also found that the content of the arguments played a very important role. Whenever a strong argument is combined with a weak argument, participants mostly base their conclusion on the strong argument and consequently ignore the conventional interpretation of *but* (and *so* and *nevertheless*). Additionally, we found an effect of axiological value, which is in line with the positive–negative asymmetry theory.

## Introduction

When people communicate with each other, they tend to follow a cooperative principle to make their message easily understood by all interlocutors (Grice, 1989). This implies to follow some rules that Grice describes as maxims. This cooperative principle allows interlocutors to derive implicatures, i.e., inferences that consist of attributing to a speaker an implicit meaning that goes beyond the explicit linguistic meaning of an utterance. Consider the following example:

(1) Some students passed the exam.

The utterance in (1) will be interpreted as “*Not all of the students passed the exam.*” If all of the students had passed the exam, (1) would still be logically true. However, the hearer can assume that the interpretation of ‘some’ as ‘not all’ holds because the speaker wants his utterance to be optimally understood by the hearer by being as informative as possible. The inference from (1) that not all the students passed the exam is an example of a conversational implicature.

There are, however, implicatures that are not derived from the cooperative principle and are therefore independent of its four maxims. They are called conventional implicatures. These implicatures are attached by convention to particular lexical items or linguistic constructions. Grice (1975) wrote the following about them:

“In some cases the conventional meaning of the words used will determine what is implicated, besides helping to determine what is said. If I say (smugly), *He is an Englishman; he is, therefore, brave*, I have certainly committed myself, by virtue of the meaning of my words, to its being the case that his being brave is a consequence of (follows from) his being an Englishman” (Grice, 1975, p. 44).

The use of the word *therefore* implies a consequence link between the two sentences. This link, however, does not contribute to the truth conditions of the sentence “*he is an Englishman*” nor of the sentence “*he is brave.*” Indeed, if a sentence ‘*p therefore q’* is true, it follows that ‘*p and q*’ is true, and therefore, that *p* is true and that *q* is true too. The contribution of *therefore* is in other words non-truth-conditional; it is not needed for the truth-conditional analysis. This idea is also expressed in the following definition by Horn (2004):

“Unlike an entailment or logical presupposition, this type of inference is irrelevant to the truth conditions of the proposition. This inference is not cancellable without contradiction, but it is detachable, in the sense that the same truth-conditional content is expressible in a way that removes (detaches) the inference. Such detachable, but non-cancellable aspects of meaning that are neither part of, nor calculable from, ‘what is said’ are conventional implicatures.” (Horn, 2004, p. 4)

The implicatures stemming from the connector *but* are classically also described as conventional implicatures. This claim will be questioned in the current paper. The materials used in our experiment consist of ‘p but q’ sentences (‘p *maar* q’ in Dutch, the language in which the experiment is carried out) in which *but* operates as a distancing contrastive connector, more specifically as an indirect one. In a distancing contrast, *but* connects two parts of a complex speech act and the second part is dissociated from the first part, without explicitly denying what is being expressed in the first part (Van Belle and Devroy, 1992). The speaker endorses or recognizes that *p* is true (Van Belle, 2003). However, *but* prevents the inference that would normally be derived from *p*. This can happen in two ways. The first possibility is that *q* contains a conclusion that contradicts the inference from *p*. Consider the following example (Van Belle, 2003):

(2) The milk is sour, but I drink it.

On the basis of *p*, one expects that the speaker will not drink the milk. However, *q* contradicts directly this expectation. This is an example where *but* operates as a direct distancing contrastive connector, sometimes also called a ‘concluding *but*’ (Van Belle and Devroy, 1992).

The second possibility is the one that is investigated in this article. In this construction *q* consists of an argument that leads to an expectation that contradicts the expectation from *p*. For example:

(3) The milk is sour, but I am thirsty.

The inference from *p* is that the speaker in (3) will not drink the milk. The inference from *q*, however, is that the speaker will drink the milk. Anscombre and Ducrot (1977; see also Van Belle, 2003; Potts, 2015) claim that the second phrase in such an indirect distancing contrast has more weight. Consequently the conclusion follows that the speaker will drink the milk.

The conclusion from a ‘p but q’ sentence can be introduced by words like *so* (*dus* in Dutch) or *nevertheless* (*toch* in Dutch). *So* and *nevertheless* also demonstrate that words might have no effect truth-conditionally, but still carry information. The word *so* elicits the inference from *q* as the conclusion. In other words, from (3), it follows:

(4) So I will drink the milk.

One can say that *so* strengthens the inference from *but*, or, stated differently, it signals that the previous information/expectation explains the next fact. It is important to notice that *so* plays no role in the truth conditions of (4). In other words, (4) is true if and only if it is true that

(5) I will drink the milk.

This truth-conditional analysis does not mean that *so* has no purpose in the sentence. It signals that what follows is causally linked with the previous information. In contrast to the previous truth-conditional analysis, the word *nevertheless* cancels the inference from *but* and elicits the inference from *p* as the conclusion from (3):

(6) Nevertheless I will not drink the milk.

As with *so*, *nevertheless* does not play a role in the truth conditions of (6), although it signals something, i.e., that what will be presented is in contrast with previous information/expectation. Indeed, (6) is true if (7) is true, and false otherwise:

(7) I will not drink the milk.

Janssens and Schaeken (2013) investigated experimentally how people understand *but*, *so*, and *nevertheless*. They presented 63 adult participants with such ‘p but q’ sentences followed by either two *so*-conclusions or two *nevertheless*-conclusions. Participants were asked what the person in the story would conclude. Janssens and Schaeken (2013) aimed to find out whether people understand *but*, *so*, and *nevertheless* as predicted by the literature on conventional implicatures, or if other factors, like the content of the sentences, were driving the interpretation. If conventional implicatures were the driving force behind the interpretation, people would choose the inference from *p* when the conclusion with *nevertheless* is asked and the inference from *q* when the *so*-conclusion is asked. The *p*- and *q*-arguments were either both sensible (i.e., they both made sense) or a combination of a sensible and an irrelevant argument. According to the account of Anscombre and Ducrot (1977) implicatures stemming from *but*, *so*, and *nevertheless* should lead to a certain conclusion, irrespective of the (relevance of the) content of the arguments. The results showed that, although people seemed to follow the conventional implicatures, the content of the arguments also greatly influenced participants’ answers. When a sensible argument was combined with an irrelevant argument, participants mostly based their conclusion on the sensible argument. Even when a combination of two sensible arguments was presented, performance was not perfect. A plausible interpretation of the imperfect performance is that the content of the arguments often prevails over the implicatures that could be drawn from the ‘p but q’ sentences. This interpretation is in line with the results of Experiment 2 in Janssens and Schaeken (2013), where participants had to justify their responses. When reasoners gave an unconventional conclusion (i.e., not in line with what is predicted on the basis of the literature about the conventional implicatures of *but*, *so*, and *nevertheless*), they tended to refer to the content of that argument. Another finding from Janssens and Schaeken (2013) was that *nevertheless* elicited more unconventional answers than *so*. They argued that this might be attributed to the fact that the *nevertheless* doesn’t actually evoke the inference from *p* as was predicted. There are, however, alternative explanations. It might be that *nevertheless* evokes the negation of the conclusion from *q*. This negation does not necessarily mean the inference from *p*. Indeed, after (3) one might for instance say “*nevertheless I’m hesitating.*” Another plausible explanation is in terms of effort. In order to reach the conventional *nevertheless*-conclusion from *p*, the implicature from *but* (i.e., the inference from *q*) has to be overruled, which seems likely to be effortful.

There are no existing theories claiming that there is or should be specific processing costs involved in processing these specific *but* implicatures. However, according to Blakemore (1987) and Iten (2005), *but* encodes a specific procedure. In the context of Relevance Theory, Blakemore (2002) developed a procedural analysis of *but*. This analysis asserts that *but* “encodes a constraint that triggers an inferential route involving contradicting and eliminating an assumption that is manifest in the context” (in Hall, 2004, p. 220). Iten (2005) refined Blakemore’s analysis of *but* and claimed “what follows (q) contradicts and eliminates an assumption that is accessible in the context.” If we try to translate these in terms of processing costs, it seems fair to argue that the contradiction and elimination procedure is requiring extra processing costs. In order to reach the conventional conclusion from a ‘p but q’ sentence as an indirect distancing contrast, one must infer the specific conclusion from *p* and the specific conclusion from *q* (which is opposite to the conclusion from *p*). Additionally, *but* implies that the second argument weighs more heavily so that the putative conclusion of *p* is eliminated and the final conclusion is inferred from *q*. As a consequence, four inferences should be made in order for this final conclusion to be reached. Stated more formally, the inference steps are the following (where *p* stands for the *p*-argument, *q* for the *q*-argument, and *r* for the conclusion you can derive from the respective arguments):

(1) p but q
(2) (p → r) but (q →¬ r)
      [=Introduction of the expected conclusion that follows from the arguments in (1)]
(3) r and ¬ r
      [=Contradiction that follows from (2)]
(4) ¬ r > r
      [=The bigger weight of the not-r conclusion, on the basis of the *but*-implicature]
(5) ¬ r
      [=Solving the contradiction in (3) by eliminating the conjunct that has the smallest weight in (4)]
(6) ∴ *So not-r is the case.*

The inference steps 2 and 4 are an expression of the implicatures attached to *but*, inference steps 3 and 5 are inferences, which are needed in order to be able to complete the reasoning process. Moreover, when the *but*-sentence is followed by *nevertheless*, the processing costs might be even higher. After the contradiction and elimination of the conclusion following the *p*-argument, encountering *nevertheless* forces the listener to undo the elimination (or eliminate and contradict the conclusion from the *q*-argument):

(1) p but q
(2) (p → r) but (q →¬ r)
      [=Introduction of the expected conclusion that follows from the arguments in (1)]
(3) r and ¬ r
      [=Contradiction that follows from (2)]
(4) ¬ r > r
      [=The bigger weight of the not-r conclusion, on the basis of the *but*-implicature]
(5) ¬ r
      [=Solving the contradiction in (3) by eliminating the conjunct that has the smallest weight in (4)]
(6) ¬¬ r [=Negation of (5) after encountering *nevertheless*]
(7) ∴ *Nevertheless r is the case.*

An alternative account might be that people reverse the inference in step (4) after being confronted with the contradiction in (3) and the word *nevertheless*. This means that step (5) also could be

(5) ¬ (¬ r > r)

The subsequent reasoning steps would be:

(6) ¬ r ≤ r
(7) ∴ *Nevertheless r is the case.*

Thus, reasoning in line with the ‘contradiction and elimination’ view of Blakemore (1987) and Iten (2005), together with the general finding from Janssens and Schaeken (2013) that drawing these implicatures doesn’t happen flawlessly, induces the possibility that processing *but* is cognitively effortful and therefore requires specific cognitive capacity. In rule-based accounts of reasoning (see e.g., Braine and O’Brien, 1991; Rips, 1994), the number of reasoning steps influences the difficulty directly, because of their working memory load. In this perspective, the extra reasoning step needed for *nevertheless*, which might involve a double negation, a well-known difficult reasoning operation (see e.g., Schroyens et al., 2001), should tap working memory resources even more, therefore making *nevertheless* more difficult than *so*. Also the alternative account of the reasoning steps for *nevertheless*, where one reverses the already made *but*-implicature, clearly demands extra resources.

Moreover, a closer look at the inference steps 2 and 4, which are an expression of the implicatures attached to *but* as a distancing contrastive connector, reveals that they have certain properties of conversational implicatures. One specific feature that characterizes conversational implicatures but not conventional implicatures is that they are cancellable. In the next paragraph we will explain that the well-known conventional implicature *but* is not immune for cancelation, which is surprising from a theoretical point of view.

First, there are sentences in which *but* connects two parts and the use of *but* creates a contrast between the two parts. For example:

(8) She is blonde, but she is intelligent.

The use of *but* in (8) elicits, in Grice’s terms (1975), the implicature that being blonde contrasts with being intelligent (at least in the speaker’s view) although this contrast is not explicitly expressed. This contrast is an indefeasible inference from *but*. The use of the word *but* implies a contrastive link between the two parts of the sentence. This link, however, does not contribute to the truth conditions of the sentence “*she is blonde, but she is intelligent.*” The previous sentence and “*she is blonde and intelligent”* are both true if *p* is true (“*she is blonde*”) and at the same time *q* is true (“*she is intelligent*”). The contrast in (8) is due to the fact that *but* comes with an implicature that *and* lacks. Since it is part of the conventions of English that *but* is used this way, Grice calls it a conventional implicature (Geurts, 2010, p. 8). Second, *but* can also be used in sentences in which the inferences from the *p*- and *q*-argument already contrast each other. Example (3) (about sour milk but being thirsty) is an example of such a *but*-sentence. The implicature from *but* indicates that the second part of the argumentation (*q*) attains more weight (Anscombre and Ducrot, 1977). The use of *but* in sentence (8) seems indeed to be in line with a classical conventional implicature, i.e., a non-cancellable implicature. However, this is not true for the use of *but* in sentence (3), which is the type that will be discussed in this paper. For example, by using *nevertheless* the implicature from *but* is canceled. *Nevertheless* denies the inference from *but* and guides the hearer or reader toward the inference from *p*. In other words, languages even have a discourse marker to signal the cancelation, namely *nevertheless*. As a consequence, the implicatures related to *but* may not be purely conventional, because they have certain features of conversational implicatures. Indeed, the differential weighting of the *p*- and *q*-argument seems not to be conventional because it is cancellable. This similarity with conversational implicatures is an important reason why the possibility arises that processing implicatures from *but* may require similar cognitive processes and capacities as conversational implicatures.

A substantial part of the experimental research on scalar implicatures has focused on the cognitive processes underlying these inferences. There is, however, no consensus in the literature with respect to the possible cognitive processing costs associated with deriving scalar implicatures. Indirect evidence suggesting that deriving scalar implicatures is cognitively effortful can be found in developmental research. Noveck (2001), among others, found that children are more logical than adults with terms such as *might* and *some*. Because children’s cognitive capacities aren’t fully developed yet, this was considered as indirect evidence that working memory capacities are involved in deriving scalar implicatures. Likewise, Bott and Noveck (2004, Experiment 4) observed that the number of pragmatic answers dropped when participants were forced to answer quicker, indicating that pragmatic inferences require processing costs. De Neys and Schaeken (2007) presented even more direct evidence. They burdened adult participants’ working memory capacity by providing them with a secondary task during performance of the scalar implicature task. When working memory was burdened, pragmatic inferences dropped by 10%. Marty et al. (2013) replicated this working memory load effect associated with computing the scalar implicature from *some* (see also Noveck and Posada, 2003; Huang and Snedeker, 2009; Dieussaert et al., 2011).

In contrast, other literature doesn’t seem to find any processing costs for scalar implicatures. For example, Marty et al. (2013) found an opposite working memory effect on numerical implicatures. Also, Feeney et al. (2004) found that working memory capacity was associated with providing the logical interpretation on infelicitous *some* statements. They argued that working memory is involved in inhibiting the pragmatic interpretation in favor of the logical one. Other evidence suggesting that there is no role for working memory was provided by Grodner et al. (2010). They showed in a visual-world study that there was no delay associated with the pragmatic inference from *some* compared to other, non-scalar expressions. Hence, the implicature generation takes place as soon as *some* is encountered, before the full sentence is processed. Similar, Heyman and Schaeken (2015) observed in a latent class analysis that working memory capacity did not explain the interindividual variability in the interpretation of infelicitous *some* statements. In sum, findings concerning the possible cognitive processing costs associated with deriving scalar implicatures are not consistent. This mixed evidence and the possibility of cancelation of the indirect distancing contrastive *but* (which gives *but* a characteristic of a conversational implicature) makes it worth looking into the processing costs underlying these specific implicatures from *but*.

Janssens et al. (2015) replicated Janssens and Schaeken (2013), but their participants were children aged 8–12. Additionally, working memory capacity was measured by means of the Listening Span task (Daneman and Carpenter, 1980). Their results were similar to the adult results in Janssens and Schaeken (2013), but children’s competence with *but* seemed worse than adults’ competence (although a direct comparison between adults and children was not made). Because children’s working memory capacity isn’t yet fully developed, this could indicate that working memory is involved in processing implicatures from *but*. However, no effect of working memory on children’s performance was found. This finding, in turn, suggests that working memory would not be involved in processing implicatures from *but* as an indirect distancing connector.

In sum, there are some good reasons to investigate whether working memory is involved in processing *but*. The primary aim of the present study is thus to examine if working memory is involved in processing the implicatures from *but*, *so*, and *nevertheless* in those cases where *but* is used as an indirect distancing contrastive connector. We will not measure working memory capacity, but we will use the same paradigm as De Neys and Schaeken (2007) in scalar implicature research. We will look at the effect of working memory load on *but*-implicature competence by imposing a secondary task on participants that burdens working memory capacity. If the implicature requires specific effortful processing, deriving the implicature should be harder when cognitive resources are burdened. We want to emphasize that pragmatic theorists and previous experimental studies have not characterized the exact nature of the alleged effortful processing. The present study focuses on the role of executive working memory resources. These resources are widely recognized as the essential component of human cognitive capacity (see e.g., Engle et al., 1999).

Apart from the main question of our study (the effect of working memory load), we aim to answer three extra questions.

First, we want to investigate if the relevance of the arguments can overrule the expectations that accompany *but*, *so*, and *nevertheless*. Previous *but*-research showed a strong effect of content with adults (Janssens and Schaeken, 2013) and children (Janssens et al., 2015). Content and context have a profound effect on many pragmatic phenomena (see e.g., Bambini et al., 2016, for an effect of context on metaphors), as with the closely related conversational implicature *some*, content and context effects are observed (see e.g., Breheny et al., 2006; Bonnefon et al., 2009, 2011; Heyman et al., 2012).

Actually, if one wants clear answers about the content-effect, Janssens and Schaeken (2013) and Janssens et al. (2015) did use a methodology that was not fully satisfying. They presented sentences with sensible arguments, and also sentences with irrelevant arguments. For instance, in a story where someone doubts whether or not he will eat chocolate, the person thinks: “*Chocolate is very tasty, but I have blond hair*.” It is clear that the second argument is in principle irrelevant with respect to the question of whether the person will eat chocolate. For sentences with an irrelevant argument, participants did choose the conclusion stemming from the sensible argument regardless of the direction suggested by *but*, *so*, or *nevertheless*. For the example above, the majority of participants did choose the conclusion “*so he will eat chocolate*,” although the combination of *but* and *so* should have made participants to infer the negation of the conclusion expected from the *p*-argument. Janssens and Schaeken (2013) interpreted the high number of these answers as a strong sign for the importance of the content. However, these ‘awkward’ sentences might have confused the participants. The complete irrelevance of one of the arguments might have canceled the differential weighting of the arguments and might have led participants to focus exclusively on the sensible argument. Therefore, in the present study a more ecologically valid measure is used to study the effect of the content. Participants are now presented with weak and strong arguments instead of, respectively, irrelevant and sensible arguments as in Janssens and Schaeken (2013). By manipulating the strength of the arguments we hope to investigate the effect of content in a more natural way. On the basis of the previous experiments, we expect strong arguments to overrule the direction suggested by *but*, *so*, or *nevertheless*.

Second, we want to exclude a simple alternative explanation for the claim of Anscombre and Ducrot (1977) that the *q*-argument has more weight. It simply might be that the last argument in a sequence always gets more weight. This alternative explanation was overlooked in previous research. To rule it out, the effect of the instruction word *but* will be assessed by comparing performance on sentences including *but* with sentences in which the arguments are simply juxtaposed. If order is important, also in simply juxtaposed sentences, the last argument should have more weight. However, given previous findings and theorizing about *but*, we predict that the *q*-argument only gets more weight in combination with *but*.

Third, we want to verify the impact of the axiological value of the arguments. Anscombre and Ducrot (1977) used the term ‘axiological value’ to describe the argumentative orientation of an argument, which is determined by a positive or negative value that can be ascribed to its content. Arguments whose axiological value is oriented toward a positive conclusion are labeled ‘positive arguments’ and their counterparts ‘negative arguments.’ For example, suppose a person who hesitates to buy a necklace. She says: “*I really like the necklace, but it is very expensive*.” In this example, the *p*-argument (liking the necklace) is the positive argument because it is oriented toward the positive conclusion (she will buy the necklace). The *q*-argument (very expensive) is the negative argument because it is oriented toward the negative conclusion (she will not buy the necklace). Janssens and Schaeken (2013; see also Janssens et al., 2014) did not find an effect of the axiological value. There were no systematic differences between the items with negative or positive arguments in their study. Therefore, we do not expect an effect of this variable. Nevertheless, in light of the importance of replication in science (Open Science Collaboration, 2015), we treat axiological value as a possible confounding variable and we add it as an extra variable in the design.

## Materials and Methods

### Participants

A total of 210 undergraduate students from the University of Leuven (Belgium) with a mean age of 19.2 participated in our experiment. They were all native Dutch speakers and received course credit in exchange for participation.

### Implicature Task

Every participant was presented with 16 short context stories, adapted from Janssens and Schaeken (2013). These stories, programmed in E-Prime 1.1, were presented on a computer and were followed half the time by two ‘p but q’ constructions and half the time by two ‘p. q’ constructions. For example (translated from Dutch):

Mom and Ella are shopping. Ella sees a lovely teddy bear lying on the shelves. She asks Mom if she can have the teddy bear. Mom is not sure.

Mom thinks: “Ella has been bad, but she lost her teddy bear.”

or

Mom thinks: “Ella has been bad. She lost her teddy bear.”

After each argumentative construction (either ‘p but q’ or ‘p. q’), the participants were told to indicate the conclusion that the person in the story would make, based on the construction of his/her utterance. They were explicitly told not to take into account the decision they themselves would make. For the example above, the *so*-conclusions they had to choose between are *“so Ella can have the teddy bear”* and *“so Ella cannot have the teddy bear.”* Next, the participants were presented with a different pair of arguments from the same type (e.g., *Mom thinks: “Ella already has a lot of teddy bears, but she’s been very good lately”*). Now, they had to judge two conclusions form the second conclusion type (e.g., *“nevertheless Ella can have the teddy bear”* and *“nevertheless Ella cannot have the teddy bear”*). Both the 16 stories and the *so*- and *nevertheless*-conclusions were presented in a random order. In the Supplementary Data Sheet 1, the materials of a concrete trial are provided. In contrast to Janssens and Schaeken (2013), we did not use irrelevant^1^ arguments but we did make a distinction between weak and strong sensible arguments. In the example above, both the *p*- and the *q*-argument are strong sensible arguments. In the same context, an example of two weak sensible arguments is *Mom thinks: “I’m in a hurry, but it is a lovely teddy bear.”*

In order to choose plausible and good arguments for our constructions, we performed two pilot studies. In a first pilot study, 16 participants were instructed to read stories in which a person was always confronted with a ‘dilemma’ (e.g., a girl received some chocolates and has to decide whether or not to eat chocolate). We asked the participants to give both an argument why a person should do something (e.g., ‘being hungry’ is an argument to eat chocolate) and an argument for why she shouldn’t (e.g., ‘being allergic to chocolate’ is an argument not to eat it). In a second pilot study we asked 16 different participants to rate the arguments that were generated in the first pilot study on a scale from 1 (very weak argument) to 7 (very strong argument). Based on these two pilot studies we created our experimental set. For both the constructions separated by a ‘period’ and the *but* constructions, there were four possible combinations of arguments: strong–strong, strong–weak, weak–strong, and weak–weak. Moreover, we also took into account the axiological value of the arguments. The argumentative orientation can be positive or negative. A negative argument (e.g., “*Ella has been bad”*) is oriented toward a negative conclusion (she cannot have the teddy bear), whereas a positive argument (e.g., “*Ella lost her teddy bear*”) is oriented toward a positive conclusion (she can have the teddy bear). This led to a 2 × 2 × 2 × 4 design (2 connectors: *but* or ‘period’ × 2 conclusion types: *so* or *nevertheless* × 2 axiological value combinations: negative-positive or positive–negative × 4 argument combinations: weak–weak, weak–strong, strong–weak, and strong–strong).

### Working Memory Load Task

We manipulated working memory load in order to determine whether the number of conventional responses would be lower when working memory is burdened. For our working memory manipulation, we used a secondary task based on the Double Task Paradigm used in De Neys and Schaeken (2007). We created four load conditions, whereby participants were presented with a matrix with three, four, or six dots. A matrix was displayed for 850 ms before each of the 16 stories and participants had to remember the position of the dots in order to reproduce them in an empty matrix. After the matrix, the short context story appeared on the screen. The participant could take as much time as they want to read the story. When they pressed the space bar, the story disappeared and the first (*but-* or ‘period’-) sentence appeared, together with the first choice between two conclusions (two *so-* or two *nevertheless*-conclusions). These two conclusions were presented under each other, preceded by a number. When the participant had indicated his or her response (by typing the number), the second (*but* or ‘period’) sentence appeared, together with the second choice between two conclusions (two *nevertheless*-conclusions if the two *so*-conclusions were presented for the first sentence, and two *so*-conclusions if the two *nevertheless*-conclusions were presented for the first sentence). After the participant indicated the response, the sentence and the conclusions disappeared. An empty matrix appeared and the participant had to reproduce the previously presented matrix. In the low load condition, participants were presented with a 3 × 3 matrix with three dots that were always horizontally or vertically positioned. The moderate load condition was similar, but the dot pattern was more complex to remember. In this condition, participants were presented with a 3 × 3 matrix with four randomly positioned dots. In the high load condition, there were six randomly positioned dots in a 4 × 4 matrix. Finally, as a control, there was a no load condition in which the participants were not presented with matrices but were simply asked to perform the implicature task.

### Procedure

The participants individually performed the task in five groups of up to 50 students at the same time. In each group, the participants were randomly assigned to the different working memory load conditions. All participants were presented with the 16 stories, followed by two questions about the conclusion. This means that every participant answered one item of every sentence type. Meanwhile the participants performed the working memory load task. The whole task lasted approximately 12 min per participant.

## Results

### Results of Working Memory Load Task

We calculated the average number of correctly reproduced dots in every load condition. In the low load condition the average number of correctly reproduced dots was 2.78 out of 3 (93%). In the moderate load condition, participants averagely reproduced 3.45 dots out of 4 (86%) correctly. Finally, in the high load condition, the average number of correctly reproduced dots was 4.31 out of 6 (72%). This means that participants performed fairly well on the dot recall task. This was important: avoiding both floor effects and ceiling effects is essential in order to expect differences in working memory load. For this reason, we removed all participants in every load condition whose performance was less than two SD’s below the average of their condition. We removed two participants from the low load condition (*n* = 60), three from the moderate load condition (*n* = 53) and one from the high load condition (*n* = 32). This left us with a total data set of 204 participants.

### Results on the *but*-Task

First, we calculated correlations between performance on the dot recall task and performance on the implicature task. In the low load condition we found a correlation of -0.005 (*p* = 0.97). In the moderate load condition, the correlation was 0.054 (*p* = 0.7) and the high load condition yielded a correlation of 0.31 (*p* = 0.089). These correlations indicate that there is no trade-off between the working memory load task and the implicature task.

For our main analyses a generalized linear mixed model with a logit link function was used (see e.g., Baayen et al., 2008; Jaeger, 2008; Bates et al., 2011). The dependent variable was the accuracy score (0 or 1; conventional or unconventional conclusion). The model fitting procedure was implemented in R using the lmer() function from the lme4 package. We increased model complexity until the best model fit was reached. Model fit was assessed through the Bayesian Information Criterion (BIC). We included a random intercept of participants in the final model (to capture the potential degree of heterogeneity of participants) and no random slopes for participants (because we expect similar effects of our variables on participants). All fixed effects variables were dummy-coded. For a complete description of the results, see the Supplementary Data Sheet 2.

**FIGURE 1:**
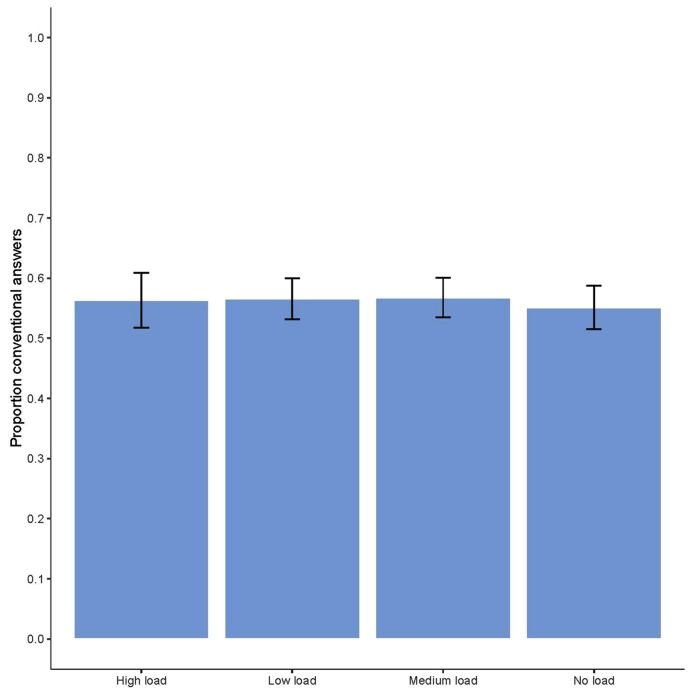
**Proportion of conventional answers for each of the four load-conditions**.

The final model includes main effects of connector, conclusion type, axiological value combination and argument combination; a two-way interaction between conclusion type and connector; and a three-way interaction between axiological value combination, argument combination and conclusion type. We did not find an effect of working memory load: there were no significant differences between the load conditions in the mean accuracy scores. These mean accuracy scores for each load condition are depicted in **Figure 1**.

**FIGURE 2:**
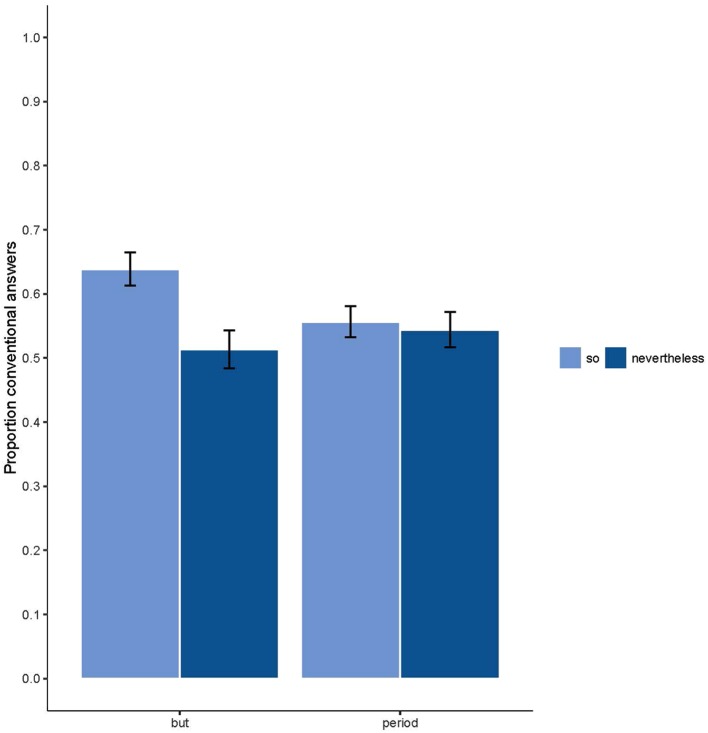
**Proportion of conventional answers as a function of connector (*but* or *period*) and conclusion type (*so* or *nevertheless*)**.

The Supplementary Data Sheet 3 displays a summary of the final model in which the intercept is compared with all other variables. *T*-tests were performed to further analyze significant effects in the model. There was a significant main effect of connector: *but* (*M* = 0.58, *SD* = 0.16) leads to more conventional answers than the ‘period’ [*M* = 0.55, *SD* = 0.15; *t*(203) = 2.312, *p* = 0.022]. Additionally, there was a significant main effect of conclusion type: *so* (*M* = 0.60, *SD* = 0.143) leads to more conventional answers than *nevertheless* [*M* = 0.53, *SD* = 0.163; *t*(203) = 6.127, *p* < 0.001]. Moreover, there was a main effect of axiological value: ‘positive–negative’ (*M* = 0.61, *SD* = 0.259) leads to more conventional answers than ‘negative–positive’ [*M* = 0.51, *SD* = 0.243; *t*(203) = 5.107, *p* < 0.001]. Finally, there was a significant main effect of argument combination [*F*(3,609) = 8.635, *p* < 0.001]. There were less conventional answers on strong–weak (*M* = 0.52, *SD* = 0.175) than on strong–strong (*M* = 0.58, *SD* = 0.193; *p* < 0.001), and on weak–strong (*M* = 0.591, *SD* = 173; *p* = 0.149). **Figure 2** displays the two-way interaction between connector and conclusion type. When the connector *but* separates the two arguments, the mean accuracy score is significantly higher for *so*-conclusions (*M* = 0.64, *SD* = 0.48) than for *nevertheless*-conclusions [*M* = 0.51, *SD* = 0.5; *t*(3262) = 7.28, *p* < 0.001]. However, when the two arguments are separated by a ‘period,’ the mean accuracy scores don’t differ significantly between *so*- and *nevertheless*-conclusions [*so*: *M* = 0.56, *SD* = 0.5; *nevertheless*: *M* = 0.54, *SD* = 0.5; *t*(3262) = 0.70, *p* = 0.48]. There are more *so*-conclusions with *but* than with ‘period’ [*but*: *M* = 0.64, *SD* = 0.48, ‘period’: *M* = 0.56, *SD* = 0.5; *t*(203) = -5.403, *p* < 0.001].

**FIGURE 3:**
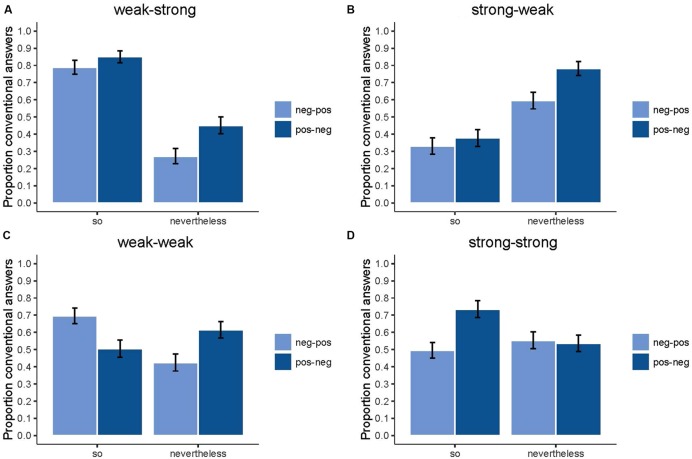
**Proportion of conventional answers as a function of conclusion type (*so* or *nevertheless*) and axiological value (*neg–pos* or *pos–neg*) for each of the four types of argument combination (*weak–strong, strong–weak, weak–weak* or *strong–strong*)**.

Concerning the three-way interaction, **Figures 3A–D** display the interactions between conclusion type and axiological value combination for each of the different levels of argument combination. In order to deal with multiple testing, Bonferroni correction was used, which set the significance cut-off at 0.000625. When a weak *p*-argument is combined with a strong *q*-argument, the axiological value combination ‘positive–negative’ leads to more accurate answers than ‘negative–positive’ for *nevertheless* [*so*/neg-pos: *M* = 0.79, *SD* = 0.41; *so*/pos-neg: *M* = 0.85, *SD* = 0.36; *t*(814) = -2.28, *p* = 0.023] (*nevertheless*/neg-pos: *M* = 0.27, *SD* = 0.45; *nevertheless*/pos-neg: *M* = 0.45, *SD* = 0.50; *t*(814) = -5.41, *p* < 0.00001]. We find the same results for the combination of a strong *p*-argument with a weak *q*-argument [*so*/neg-pos: *M* = 0.33, *SD* = 0.47; *so*/pos-neg: *M* = 0.38, *SD* = 0.49; *t*(814) = -1.39, *p* = 0.17] [*nevertheless*/neg-pos: *M* = 0.60, *SD* = 0.49; *nevertheless*/pos-neg: *M* = 0.78, *SD* = 0.41; *t*(814) = -5.86, *p* < 0.00001]. When two arguments of the same strength are presented, we see reverse patterns for strong–strong and weak–weak. In both these cases, it depends on the conclusion type whether ‘positive–negative’ or ‘negative–positive leads to more accurate answers. When both arguments are weak, the axiological value combination ‘negative–positive’ leads to more accurate answers than ‘positive-negative’ for *so*-conclusions, but to less accurate answers for *nevertheless*-conclusions [*so*/neg-pos: *M* = 0.70, *SD* = 0.46; *so*/pos-neg: *M* = 0.51, *SD* = 0.50; *t*(814) = 5.68, *p* < 0.00001] [*nevertheless*/neg-pos: *M* = 0.42, *SD* = 0.49; *nevertheless*/pos-neg: *M* = 0.62, *SD* = 0.49; *t*(814) = -5.56, *p* < 0.00001]. When both the *p*- and *q*-argument are strong arguments, we find the reverse pattern, with the exception that the difference for the *nevertheless*-conclusions is not significant [*so*/neg-pos: *M* = 0.50, *SD* = 0.50; *so*/pos-neg: *M* = 0.74, *SD* = 0.44; *t*(814) = -7.27, *p* < 0.00001] [*nevertheless*/neg-pos: *M* = 0.55, *SD* = 0.50; *nevertheless*/pos-neg: *M* = 0.54, *SD* = 0.50; *t*(814) = 0.49, *p* = 0.62).

Additionally, we performed two *post hoc* exploratory analyses, one on the asymmetric conditions (weak–strong and strong–weak) and one on the symmetric conditions (weak–weak and strong–strong). This was inspired by the asymmetry that is visible in **Figures 3A,B** between the strong–weak and the weak–strong combination. We performed the same analysis as the original one. Hence, the dependent variable was again accuracy; we included a random intercept of participants in the final model and no random slopes for participants. Again, all fixed effects variables were dummy-coded. For a complete description of the results, see the Supplementary Data Sheet 4. Here, we want to highlight two important points of these *post hoc* exploratory analyses. First, also for these models, adding working memory does not lead to an improvement of the model fit, neither when we add working memory as a main effect nor when working memory is part of an interaction. Second, the interaction between conclusion type and argument combination was significant for both extra analyses, but the pattern was different. For the asymmetric conditions, the difference between *so* and *nevertheless* was significant for the weak–strong combination [*so*: *M* = 0.82, *SD* = 0.21; *nevertheless*: *M* = 0.36, *SD* = 0.29, *t*(203) = 18.069, *p* < 0.001] and also for the strong–weak combination, but in the other direction [*so*: *M* = 0.35, *SD* = 0.29; *nevertheless*: *M* = 0.69, *SD* = 0.24, *t*(203) = -12.016, *p* < 0.001]. For the symmetric conditions, the difference between *so* and *nevertheless* was in both conditions significant, but now *so* was always easier than *nevertheless* [for the strong–strong combination, *so*: *M* = 0.62, *SD* = 0.24; *nevertheless*: *M* = 0.55, *SD* = 0.26, *t*(203) = 3.116, *p* < 0.02; for the weak–weak combination, *so*: *M* = 0.60, *SD* = 0.26; *nevertheless*: *M* = 0.52, *SD* = 0.25, *t*(203) = 3.506, *p* < 0.001].

## Discussion

The primary aim of the present study was to investigate if working memory is necessary during the processing of the implicatures from *but*. There were four working memory load conditions in order to explore whether a higher burden on working memory capacity would significantly decrease the number of conventional answers. Additionally, our experimental design enabled us to investigate the effect of three other variables. First, we made a distinction between weak and strong arguments instead of irrelevant and sensible arguments, which provides a more reliable measure of the effect of content of the arguments. Second, we made a comparison between the connectors *but* and ‘period’ to explicitly look at the effect of *but*. Third, we manipulated the axiological value of the arguments, to control if the null effect of previous findings could be replicated in a better designed study.

Our best fitting model included a two-way interaction between conclusion type (*so* and *nevertheless*) and connector (*but* and ‘period’), a three-way interaction between conclusion type, argument combination and axiological value combination and the main effects of all these variables. We will discuss the consequences of these results for our different hypotheses.

### The Role of Working Memory

The working memory load variable was not included in the best fitting models for the data. This finding is line with the results in Janssens et al. (2014), who measured working memory capacity in children and found no relation with their performance on the implicature task. This suggests that processing the implicatures from *but*, *so*, and *nevertheless* used in ‘p but q’ sentences as indirect distancing contrasts happens effortlessly without involvement of working memory. In what follows, we will place this putative conclusion into perspective, by discussing four aspects.

First, this null effect might have consequences for the theoretical underpinnings of *but*. A procedural analysis of *but* (with the ‘contradiction and elimination’ principle; see Blakemore, 1987; Iten, 2005) seems to suggest that processing *but* is effortful. Since our results showed that deriving these implicatures does not seem to be effortful, one can doubt this ‘contradiction and elimination’ view. Hall (2004, 2007) already postulated that the clause introduced by *but* does not eliminate an assumption, but merely introduces an argument that points in a different direction. She explicitly says “the implication of the second clause … does not entirely seem to replace the implication of the first clause … It just has more weight, and this is all that follows from the constraint I’m proposing.” (Hall, 2004, p. 229). The proposal of Hall is less demanding with respect to working memory costs, because the elimination is not part of it. Her argumentation might make it more understandable why we did not find that processing *but* is cognitively effortful. In addition, one could claim that this prediction can also be derived from Grice’s theory. For example, Moeschler (2012) wrote:

“This is a very important point in Grice’s definition of a conversational implicature, because only conversational implicatures are supposed to be worked out. When an implicature is automatically triggered, through a reference to the meaning of a word, the implicature is conventional.” (Moeschler, 2012, p. 417).

Second, we investigated only one type of *but*-sentences (with *but* indicating an indirect distancing contrast). One could argue that the implicatures from *but* investigated in this paper are not purely conventional. One of the major characteristics of a conventional implicature is that this inference is not cancellable without contradiction (see Horn, 2004). However, the more heavily weighting of the *q*-argument in the indirect distancing contrastive ‘p but q’ sentences can be canceled, for instance when *nevertheless* introduces the conclusion (and sometimes even when *so* introduces the conclusion, as our participants clearly did). Hence we are not making any claims about working memory involvement in other types of *but* or in conventional implicatures in general.

Third, the correlations between the number of correctly reproduced dots and accuracy on the implicature task did not support the idea of a trade-off between the two tasks. This finding is also in line with the conclusion that working memory doesn’t seem to influence the implicatures investigated in this study. However, we also found that the percentages of correctly recalled dots were highest in the low load condition and lowest in the high load condition. One might argue that, if processing these specific implicatures from *but*, *so*, and *nevertheless* truly happens automatically, then we should expect these percentages on the dot recall task to be equal for each load condition. It can be argued that participants might have invested an equal amount of working memory capacity into the implicature task and that this goes at the expense of performance on the dot recall task (especially in the high load condition). However, this suggestive explanation seems unlikely since the percentages of correctly reproduced dots were fairly high, so we would have at least expected a difference with the no load condition (which was not found). Moreover, the moderate load condition in our study corresponds to the high load condition in the original De Neys and Schaeken (2007) study. This means that our high load condition was truly highly burdening and therefore a lower percentage of correctly reproduced dots compared to the other two load conditions is not surprising.

Fourth, the observed null effect of working memory must be seen in a wider picture. There is a difference between the task in the current paper on the one hand and for instance the task of De Neys and Schaeken (2007) on scalars on the other hand. In the latter study, there is no correct answer. *Some* refers to an indeterminate amount; therefore *some* is compatible with *some and not all*, but also with *all*. In other words, *some* is ambiguous and when interpreting *some*, participants have to decide, based on contextual information, to go either for the reading with or for the one without the scalar implicature. However, *but*, *so* and *nevertheless* are not ambiguous in the way *some* is ambiguous: their meaning is clear. One only has some freedom in taking care of the weights of the arguments when coming to an interpretation. It might well be that working memory resources play a different role in these cases. This point can be made clear by using the framework offered by Chemla and Singh (2014a,b). In their stimulating review study, Chemla and Singh provide evidence that the derivation process of scalar implicatures is indeed costly. However, their careful analysis identified different possible derivation processes and it is not clear what in the derivation process of a scalar implicature creates an extra cost. The research of Marty and Chemla (2013) suggests that the processing of the alternatives is not the most effortful part in the derivation of implicatures, but that the decision step (the choice between the two readings) is the costly process (however, see van Tiel and Schaeken, 2016). The current data can be interpreted as in line with this hypothesis. Indeed, there is no need to disambiguate between two readings when interpreting a *but*-sentence, because there is just one reading. One only has to play with the weights of the arguments. Nevertheless, we want to refrain from too strong conclusions about working memory involvement, because our study on its own does not allow to conclude that working memory is not involved at all.

### The Role of Arguments Order

The two-way interaction in the model (between conclusion type and connector; see **Figure 2**) is informative with respect to our hypothesis about the effect of order. Indeed, it provides evidence that the conclusion from *so* leads to more conventional answers than the conclusion from *nevertheless*, at least when *but* separates the *p*- and *q*-argument. This means that *but* is interpreted in line with the expectations expressed in the introduction and contributes to the understanding of *so* and *nevertheless*. The inference from *but* directs the reader toward the conclusion from the *q*-argument and the use of *so* following a *but*-sentence confirms and strengthens this conclusion. However, *nevertheless* requires the reader to overrule the inference from *but* in favor of the conclusion from *p*. When a ‘period’ separates the *p*- and *q*-argument, there is no indication which of the two arguments has more weight and therefore what conclusion is the expected one. Consequently, there is no significant difference in the number of conventional answers between *so*-conclusions and *nevertheless*-conclusions in the period-condition. Moreover, there are less conventional *so*-conclusions in the period-condition than in the *but*-condition. Therefore and as predicted, we seem to be able to rule out the alternative explanation that the *q*-argument gets more weight simply because it is the last given argument. However, it’s still possible that some reasoners interpret *nevertheless* as a word that gives freedom with respect to making the inference from the *p*- or from the *q*-argument. We will come back to this last issue in the paragraph on the role of content and axiological value.

When people interpret a sequence of sentences, they want to relate portions of the text or sentences. Rhetorical relations (also called discourse relations or coherence relations) have been proposed as an explanation for the construction of coherence in discourse (see e.g., Lascarides and Asher, 1993; Asher and Lascarides, 2003). Examples of rhetorical relations are condition, motivation, purpose, and volitional cause. *But* makes the rhetorical relationship in the *but*-condition explicit (contrast), but reasoners can of course infer a rhetorical relationship themselves in the period-condition. Given the fact that we had combinations of (pretested) sensible arguments in the current experiment, although with a different orientation, it seems fair to argue that most participants have inferred the contrast-rhetorical relation. Therefore, the difference between the *but*-conditions and period-condition for the *so*-conclusions is really convincing evidence in favor of the hypothesis that it is *but* that leads to a different weighting. Because a signaled rhetorical relation (by means of *but*) might be easier to construct than an unsignaled one (in the period-condition), we only used the period-condition as a control for the temporal order hypothesis and not for the working memory involvement hypothesis.

### The Role of Content and Axiological Value

The three-way interaction, although difficult to interpret, seems to be informative with respect to our hypotheses about the role of content and the axiological value. We had not anticipated an effect of axiological value combination. The variable was basically added as a control variable, although we wanted to verify its null effect found in previous studies. The role of the argument combination variable, however, was not unexpected. Previous studies (albeit in a maybe methodologically less precise way) already gave evidence for the effect of content.

**Figure 3C** depicts the situation in which two weak arguments are presented. It can be argued that this is not an obvious situation. Compared to weak–strong and strong–weak, none of the two arguments stands out over the other. Compared to strong–strong, the weak–weak construction only contains weak arguments and it may be less clear which inference stems from these weak arguments. **Figure 3C** shows that in this weak–weak situation, people make more correct inferences from a positive argument than from a negative argument for both *so* and *nevertheless.* This can be deduced from the fact that the axiological value combination ‘negative–positive’ leads to more conventional *so*-conclusions than ‘positive–negative’ and the opposite applies for *nevertheless.* Since conventional *so*-conclusions are inferred from the *q*-argument and *nevertheless*-conclusions from the *p*-argument, this means that positive arguments facilitate the conventional conclusion in weak–weak situations.

The same seems to hold for other less obvious situations with different argument combinations. We found that, overall, *nevertheless*-conclusions elicited more unconventional conclusions than *so*-conclusions and for these *nevertheless*-conclusions a positive argument seems to facilitate the conventional conclusion compared to a negative argument as well. This can be seen in **Figure 3A** (weak–strong) and **Figure 3B** (strong–weak). However, this does not hold for the *nevertheless*-conclusions when *p* and *q* are both strong arguments. In those sentences, there was no significant difference between ‘positive–negative’ and ‘negative–positive.’ The fact that in general *nevertheless* is better understood with a positive *p*-argument might be seen as evidence in favor of the claim that reasoners are able to interpret *nevertheless* as pointing toward the conclusion from the *p*-argument, at least when the circumstances are ideal, i.e., when not too much processing is required and a preferred axiological value construction is used.

When we look at the *so*-conclusions, a reverse pattern seems to emerge. In the strong–strong (**Figure 3D**) situations, the axiological value combination ‘positive–negative’ leads to significantly more conventional *so*-conclusions than ‘negative–positive,’ which implies that a negative argument facilitates the conventional conclusion in these situations. This difference between ‘positive–negative’ and ‘negative–positive’ is not significant for the *so*-conclusions in the strong–weak situations (**Figure 3B**). This can be explained by the fact that this is the least obvious *so*-conclusion to make, since it requires the reader to ignore a strong argument in favor of a weak argument. This explanation, however, does not match with the absence of a difference in the weak–strong situation. Here the reader has in principle only an easy job to do, that is, ignore a weak argument in favor of a strong one.

These results seem to be somewhat in line with the positive–negative asymmetry theory (Peeters and Czapinski, 1990; Taylor, 1991; Rozin and Royzman, 2001). Lewicka (1988, 1998) has demonstrated that this theory can account for human deviations from normative models of reasoning (see e.g., Verschueren et al., 2006). According to the theory, human information processing bears the marks of a general tendency to have greater subjective necessity associated with avoiding negative outcomes than with obtaining positive outcomes. The general trend we observe is that *so*-conclusions are easier with the negative–positive form, while the opposite is true for *nevertheless*. In both cases this means that the conclusion is easier when it is based on the positive argument (*q* in the case of *so* and *p* in the case of *nevertheless*). We do not have a clear explanation yet why and how exactly this effect interacts with the strength of the arguments. Nevertheless, our results indicate that emotional factors can penetrate the interpretation process in a very subtle yet influential way. The current experiment seems to show that even non-intrusive *but*-sentences change depending on whether people read a positive or negative *p*- or *q*-argument. However, since the effect of axiological value combination was unexpected, the proposed analysis remains suggestive. Therefore, replication and variation studies are mandatory in order to firmly establish this positive–negative effect and the interaction with the strength of the arguments.

The two extra analyses not only confirmed the null-effect of working memory, they also revealed an interesting extra finding. For the weak–strong combination, we observed more expected *so*-conclusions than *nevertheless*-conclusions; for the strong–weak combination, we observed more expected nevertheless-conclusions than so-conclusions. This interaction can be phrased differently, namely, for both combinations participants just preferred conclusions on the basis of the strong argument, which is the *q*-argument for the weak–strong combination and the *p*-argument for the strong–weak combination. This seems to indicate that participants were strongly driven by the strength of the arguments in the asymmetric condition. This observation is in line with Hall’s (2004; 2007) theory. She claims that the *q*-argument does not eliminate an assumption, but merely announces an argument that points in a different direction. The *q*-argument has more weight and is preferred over the *p*-argument, but when the content of the *p*-argument allows it, a conclusion can be drawn from *p*. Hence, Hall (2004, 2007) indirectly emphasizes the significance of the content of the arguments.

## Conclusion

This experiment showed that, when presented with *but*-constructions indicating an indirect distancing contrast, people tend to attribute more weight to the *q*- than the *p*-argument. The experiment also showed that participants under a high working memory load did not perform significantly different from participants under a low working memory load or whose working memory was not burdened at all. Concerning the different conclusion types, we found that more unconventional answers are given when participants have to infer the *nevertheless*-conclusion than when they have to infer the *so*-conclusion. We also found that the content of the arguments played a very important role. Whenever a strong argument is combined with a weak argument, participants mostly base their conclusion on the strong argument and consequently ignore the conventional interpretation of *but* (and *so* and *nevertheless*). Hence, even sensible arguments can get annulled simply because they are weak and measured against a stronger argument. The latter effect might be modulated by the axiological value of the arguments. In other words, the strength or perceived relevance of the *p*- and *q*-arguments can override the expectations elicited by *but*, *so* and *nevertheless*: content and context are important forces during our interpretation process.

## Author Contributions

All authors contributed to this article, both substantively and formally. LJ and WS prepared the experiment. LJ performed the experiment and did the statistical analysis. WS did the data interpretation. LJ was the driving force in writing the first version and WS of the final version. All authors contributed equally to the editing of the manuscript and approved the final version of the manuscript.

## Conflict of Interest Statement

The authors declare that the research was conducted in the absence of any commercial or financial relationships that could be construed as a potential conflict of interest.

## Footnotes

1 Note that ‘irrelevant’ is labeled as ‘absurd’ in the original Janssens and Schaeken (2013) study.
