# Supplementary material for: ‘But’ Implicatures: A Study of the Effect of Working Memory and Argument Characteristics
Source: Front Psychol. 2016 Nov 8;7:1520. doi: 10.3389/fpsyg.2016.01520 (PMC5100440; doi:10.3389/fpsyg.2016.01520)
Supplement: Supplementary file 1 [file Data_Sheet_1.pdf]

frontiers
Supplementary Material

Supplemental Data Sheet: Additional Information

'But' Implicatures: A Study of the Effect of Working Memory and Argument Characteristics

Leen Janssens and Walter Schaeken*

*** Correspondence:** walter.schaeken@ppw.kuleuven.be

Supplemental Data Sheet 1: The 16 stories with the accompanying *so*- and *nevertheless*-conclusions in the order of a concrete trial.

Neg first: the *p*-argument was negative*, Pos first: the p*–argument was positive
Neg second: the *q*-argument was negative*; Pos* second: the *q*-argument was positive
Weak: the argument was weak*; Strong:* the argument was strong
_: the arguments were separated by a period; *but:* the arguments were connected by *but*

1. NegWeak_PosStrong
Before Lennert and Vincent go to sleep Dad comes by to give them a goodnight kiss. The boys ask Dad if he wants to tell them a horror bedtime story. Dad doubts whether he would do so.

Dad tells Mom: I want to go to sleep myself. The boys behaved well today.
So, I' won't tell them a horror story.
So, I will tell them a horror story.

Dad tells Mom: There's football on TV. The boys love stories
Nevertheless, I won't tell them a horror story.
Nevertheless, I will tell them a horror story.

2. PosStrong_but_NegWeak
The junior soccer team of Haasrode plays a match against the junior soccer team of Bierbeek. Two spectators are discussing the potential chances of victory for the team from Haasrode. Mark doubts if the soccer team from Haasrode will win.

Mark says: The soccer team of Haasrode is the best team in the ranking, but they are the away-team.
So, they have little chance to win.
So, they have lots of chance to win.

Mark says: The soccer team of Haasrode is the best team in the ranking, but the match is played on a very bad soccer field.
Nevertheless, they have little chance to win.
Nevertheless, they have lots of chance to win.

3. NegWeak_but_PosStrong
The Verhulst family wants to spend an evening playing board games. Everyone participates. Only the oldest son, Alexander, is still unsure whether he will play or not.

Alexander thinks: I'm angry with Dad, but they play my favourite game.
So, I will play board games with my family.
So, I won't play board games with my family.

Alexander thinks: I always lose, but I like to take part in family activities.
Nevertheless, I won't play board games with my family.
Nevertheless, I will play board games with my family.

4. NegWeak_PosWeak
Mom and Ella are shopping. Ella sees a lovely teddy bear lying on the shelves. She asks Mom if she can have the teddy bear. Mom is not sure.

Mama thinks: Ella's birthday is within a month. I have to make it up with her.
So, Ella can have the teddy bear
So, Ella cannot have the teddy bear.
Mama thinks: I'm in a hurry. It's a beautiful teddy bear.
Nevertheless, Ella can have the teddy bear
Nevertheless, Ella cannot have the teddy bear.

5. PosWeak_NegWeak
Ellen goes to a concert tonight and asks Katrien if she would go with her. Katrien doubts whether she will go to the concert.

Daisy tells Ellen: I need to start working a bit later the following morning. I don't know the people Ellen goes with.
So, I won't go to the concert.
So, I will go to the concert.

Daisy tells Ellen: I bought new earplugs. I'm tired.
Nevertheless, I will go to the concert.
Nevertheless, I won't go to the concert.

6. PosWeak_but_NegStrong
Laurens has to find sponsors for an event he is organizing. He asks his neighbour, Thomas, if he wants to go along with him to search for sponsors in the neighbourhood. Thomas is not sure whether or not to help Laurens.

Thomas thinks: I have nothing better to do, but I'm sick.
So, I will join Laurens to find sponsors.
So, I won't join Laurens to find sponsors.

Thomas thinks: I myself have not found a single sponsor, but I have other things to do.

Nevertheless, I won't join Laurens to find sponsors.

Nevertheless, I will join Laurens to find sponsors.

7. NegStrong_PosStrong

Each year in the H. Hart Heverlee high school a teacher is awarded with the title of 'teacher of the year'. There is speculation among the students who will get the title this year. Bob, a boy from that school, wonders whether Mr. Van Damme will get the title.

Bob says: He gives little explanation during his lessons. He helps students when they have problems.

So, I think he won't receive the title of 'teacher of the year'.

So, I think he will receive the title of 'teacher of the year'.

Bob says: He tends to favour certain students. He is good at teaching.

Nevertheless, I think he will receive the title of 'teacher of the year'.

Nevertheless, I think he won't receive the title of 'teacher of the year'.

8. PosStrong_but_NegStrong

Maggie, the cat of the Mertens family, gave birth to four kittens. The Mertens family wants to give the kittens to the Peeters family. The Peeters family is unsure if they will keep the kittens.

Mommy says: We're all crazy about cats, but dad is allergic to cats.

So, we will keep the kittens.

So, we won't keep the kittens.

Mom says: We wanted already for a long time to buy a cat, but in fact it turns out we do not like cats.

Nevertheless, we won't keep the kittens.

Nevertheless, we will keep the kittens.

9. PosStrong_NegWeak

It's Christmas. The De Corte family bought a Christmas tree and they want to decorate it. Mom is in doubt whether or not to let her youngest daughter, Sarah, help.

Mom explains: Sarah likes to do things together with me. She drops a lot of balls.

So, Sarah can help.

So, Sarah cannot help.

Mom explains: Sarah likes decorating. She is too small to reach the top of the tree.

Nevertheless, Sarah can help.

Nevertheless, Sarah cannot help.

10. NegStrong_but_PosStrong

Miss Clara returns the exams that she corrected yesterday. Luk wonders if he will have a good grade.

Luk thinks: I did not understand the subject matter, but I could solve all the questions.

So, I think I will have a good grade.

So, I think I won't have a good grade.

Luk thinks: I had an arduous struggle over the questions, but I had prepared well for them.
Nevertheless, I think I won't have a good grade.
Nevertheless, I think I will have a good grade.

11. PosStrong_NegStrong
During the holidays, Saartje is staying with her grandmother for a couple of days. Saartje is sitting outside in the garden when her grandmother brings her a glass of lemonade. Saartje is unsure whether or not to drink the lemonade.

Saartje says: I'm thirsty. I don't like lemonade.
So, I will drink lemonade.
So, I won't drink lemonade.

Saartje says: Lemonade is my favourite beverage. The lemonade has expired.
Nevertheless, I will drink lemonade.
Nevertheless, I won't drink lemonade.

12. PostiefWeak_but_NegWeak
Pieter's friends decide to have a hamburger for lunch. Pieter is unsure whether or not to join them.

Pieter says: I have enough money, but last week I already ate a hamburger.
So, I won't join my friends for their hamburger-lunch.
So, I will join my friends for their hamburger-lunch.

Pieter says: I do not feel like eating my sandwiches, but I only have one hour left.
Nevertheless, I won't join my friends for their hamburger-lunch.
Nevertheless, I will join my friends for their hamburger-lunch.

13. NegWeak_but_PosWeak
Santa Claus has brought many sweets this year. Evelien received lots of chocolates. She doubts whether she would eat chocolates or not.

Evelien thinks: Chocolates make you fat, but they expire soon.
So, I won't eat chocolates.
So, I will eat chocolates.

Evelien thinks: I might get stomachache, but I've got plenty of chocolates.
Nevertheless, I will eat chocolates.
Nevertheless, I won't eat chocolates.

14. NegStrong_PosWeak
Klaas is playing on the playground at school. He sees how Tim, his best friend, beats another child. Klaas doubts whether he should tell the teacher.

Klaas says: Tim is my best friend. The teacher will reward me if I tell him.
So, I will tell the teacher that Tim someone pounded.
So, I won't tell the teacher that Tim someone pounded.

Klaas says: I don’t want to be a tattletale. Tim has already pounded me.
Nevertheless, I will tell the teacher that Tim someone pounded.
Nevertheless, I won’t tell the teacher that Tim someone pounded.

15. PosWeak_NegStrong
Marie has bought a plant for Mom’s birthday. She wants the plant to live as long as possible. Marie doubts whether this will succeed.

Marie thinks: The plant is kept inside. The plant gets no sunlight.
So, I am convinced that the plant won’t stay alive.
So, I am convinced that the plant will stay alive.

Marie thinks: I can take away the rotten leaves immediately. The plant is not perfectly suited for this environment.
Therefore, I am convinced that the plant won’t stay alive.
Therefore, I am convinced that the plant will stay alive.

16. NegStrong_but_PosWeak
Mom and Jens are going to the dentist. Mom asks Robbe if he will come along. Robbe doubts whether he would go to the dentist or not.

Robbe thinks: I've just been to the dentist, but Mom would like me to go.
So, I won’t go to the dentist.
So, I will go to the dentist.

Robbe thinks: My insurance has expired, but I always get a lollipop from the dentist.
Nevertheless, I will go to the dentist.
Nevertheless, I won’t go to the dentist.

Supplemental Data Sheet 2: The percentage appropriate conclusions in the different conditions.

		SO								NEVERTHELESS							
		POS-NEG				NEG -POS				POS- NEG				NEG- POS			
		WW	SS	SW	WS	WW	SS	SW	WS	WW	SS	SW	WS	WW	SS	SW	WS
BUT	NL	56	85	49	90	81	54	36	83	56	42	75	36	46	58	61	25
	LL	63	77	37	83	73	57	38	80	77	50	73	43	40	47	62	30
	ML	55	68	34	89	75	53	30	79	70	43	77	45	42	55	57	21
	HL	56	81	31	78	75	56	63	78	63	66	78	41	38	53	59	19
PERIOD	NL	41	68	37	86	54	36	25	73	53	58	69	42	43	60	63	29
	LL	38	65	40	83	63	55	18	82	61	53	85	50	44	58	63	37
	ML	53	74	26	81	66	42	36	81	60	70	91	51	43	58	49	26
	HL	41	75	47	88	72	44	31	72	44	50	72	53	41	56	59	22

NL is No Load, LL is Low Load, ML is Moderate Load, and HL is High Load. WW is the weak-weak construction, SS the strong-strong, WS is the weak-strong and SW the strong-weak.

Supplemental Data Sheet 3: The parameter estimates for the final model (main analysis).

**Predictor**	**Estimate**	**Standard Error**	***Z***	***P***
Intercept	0.20	0.11	1.75	.080
Period	0.42	0.08	5.32	<.001***
Nevertheless	0.04	0.15	0.24	.810
SW	0.74	0.15	5.0	<.001***
WS	1.41	0.16	8.81	<.001***
WW	0.90	0.15	5.99	<.001***
pos-neg	1.10	0.15	7.19	<.001***
period x nevertheless	0.56	0.11	5.17	<.001***
nevertheless x SW	0.93	0.21	4.48	<.001***
nevertheless x WS	2.69	0.22	12.14	<.001***
nevertheless x WW	1.45	0.21	6.98	<.001***
nevertheless x pos-neg	1.17	0.21	5.58	<.001***
SW x pos-neg	0.88	0.21	4.11	<.001***
WS x pos-neg	0.66	0.24	2.73	.006**
WW x pos-neg	1.96	0.21	9.15	<.001***
nevertheless x SW x pos-neg	1.88	0.30	6.19	<.001***
nevertheless x WS x pos-neg	1.57	0.32	4.89	<.001***
nevertheless x WW x pos-neg	2.85	0.30	9.63	<.001***

S=strong, W=weak
pos=positive, neg=negative
*p<.05, **p<.01, ***p<.001

Supplemental Data Sheet 4a: The parameter estimates for the final model of the asymmetric conditions (additional analyses).

**Predictor**	**Estimate**	**Standard Error**	***Z***	***P***
Intercept	0.6121	0.13	4.857	<.001***
Period	0.2591	0.12	-2.213	<.05 *
Nevertheless	0.9104	0.17	5.392	<.001***
WS	2.1269	0.17	12.836	<.001***
period x nevertheless	0.4807	0.16	2.997	<.005***
nevertheless x WS	-3.5701	0.23	-15.634	<.001***
nevertheless x pos-neg	0.7149	0.22	3.254	<.005***

S=strong, W=weak
pos=positive, neg=negative

Supplemental Data Sheet 4b: The parameter estimates for the final model of the symmetric conditions (additional analyses).

**Predictor**	**Estimate**	**Standard Error**	***Z***	***P***
Intercept	0.25337	0.12	2.117	<.05*
Period	-0.54639	0.11	-5.082	<.001***
WW	0.90180	0.15	5.975	<.001***
Pos-neg	1.10497	0.15	7.158	<.001***
period x nevertheless	0.61475	0.15	4.137	<.001***
nevertheless x WW	-1.45047	0.21	-6.927	<.001***
nevertheless x pos-neg	-1.17745	0.21	-5.576	<.001***
WW x pos-neg	-1.96470	0.22	-9.078	<.001***
Nevertheless x pos-neg x WW	2.85067	0.30	9.535	<.001***

W=weak
pos=positive, neg=negative
